# Physical characterization of fault rocks within the Opalinus Clay formation

**DOI:** 10.1038/s41598-022-08236-7

**Published:** 2022-03-14

**Authors:** Luis Felipe Orellana, Christophe Nussbaum, Luiz Grafulha, Pierre Henry, Marie Violay

**Affiliations:** 1 Laboratory of Experimental Rock Mechanics, IIC-ENAC, École Polytechnique Fédérale de Lausanne, Lausanne, Switzerland; 2 Federal Office of Topography (Swisstopo), Wabern, Switzerland; 3 Scientific Centre of Optical and Electron Microscopy, ETH Zürich, Zurich, Switzerland; 4 Centre Européen de Recherche Et d’Enseignement Des Géosciences de L’Environnement, Aix-Marseille Université, Marseille, France; 5 Now Department of Mining Engineering, FCFM - Universidad de Chile, Santiago, Chile; 6 Now Advanced Mining Technology Center (AMTC), FCFM - Universidad de Chile, Santiago, Chile

**Keywords:** Geology, Tectonics

## Abstract

Near-surface disposal of radioactive waste in shales is a promising option to safeguard the population and environment. However, natural faults intersecting these geological formations can potentially affect the long-term isolation of the repositories. This paper characterizes the physical properties and mineralogy of the internal fault core structure intersecting the Opalinus Clay formation, a host rock under investigation for nuclear waste storage at the Mont Terri Laboratory (Switzerland). We have performed porosity, density, microstructural and mineralogical measurements in different sections of the fault, including intact clays, scaly clays and fault gouge. Mercury intrusion porosimetry analysis reveal a gouge that has a pore network dominated by nanopores of less than 10 nm, yet a high-porosity (21%) and low grain density (2.62 g/cm^3^) when compared to the intact rock (14.2%, and 2.69 g/cm^3^). Thus, a more permeable internal fault core structure with respect to the surrounding rock is deduced. Further, we describe the OPA fault gouge as a discrete fault structure having the potential to act as a preferential, yet narrow, and localized channel for fluid-flow if compared to the surrounding rock. Since the fault gouge is limited to a millimetres-thick structure, we expect the barrier property of the geological formation is almost not affected.

## Introduction

Due to the inherent low permeability of shales^1,2^, they have been recognized in several countries as one of the most suitable candidates for the storage of nuclear waste in deep geological repositories^3,4^. However, it remains unclear how faults in shales might weaken the isolation of radioactive contamination from the environment and population.

Fault zones are complex, anisotropic, and heterogeneous discontinuities cutting the upper Earth’s crust^5^. Extensive research has shown that their architecture (e.g. lithology, fault zone geometry, spatial variability), their mechanics (e.g. fault displacement, fluid-rock interactions), and their fluid-transport properties (e.g. permeability, porosity) are inter-related parameters governing the fault deformation processes^5–8^. For instance, a classical yet not general model for faults in crystalline rocks usually illustrates a low-permeability (10^–18^–10^–22^ m^2^) clay-rich fault gouge core surrounded by a higher-permeability damage zone^6,9,10^. In this configuration, fault cores can then act as a barrier^11–13^ or as mixed conduit-barrier for fluids^14–16^. Therefore, faults can exert a strong control on the pore pressures and effective stresses, but also the migration of fluids in geological formations^7^.

This paper investigates the internal fault structure mineralogy, physical properties, and associated fluid-flow regime governing faults in shales. As the capacity for fluid transport is related to the connected pore structure and faults, the research herein comprises a study of the pore structure of clay-bearing fault-rocks within the Opalinus Clay formation, a potential host rock for nuclear waste storage in Switzerland^17–19^. For this purpose, we have conducted mineralogical and microstructural analysis, and we have characterized the porosity, grain density and pore structure of more than 60 samples obtained from the internal fault structures of the Main Fault, a clay-bearing fault crosscutting the Mont Terri Laboratory. Further, we discuss the flow distribution capabilities within the internal fault structures using the laboratory results integrated to empirical models of permeability. Finally, we discussed the role of clay-rich fault gouges in the Main Fault as a localized, discontinuous, and discrete pathway for fluid flow.

### Geological context

Situated near St-Ursanne in the canton of Jura, the Mont Terri Laboratory (MTL) is the underground research infrastructure devoted to the study of deep-nuclear waste storage repositories in the Opalinus Clay (OPA) formation in Switzerland^17,18^. A detailed description on the local geological setting of the Opalinus Clay formation and its surroundings can be found in Nussbaum et al. (2011). The MTL will not be the final location for nuclear waste, yet it provides a unique opportunity to foreseen potential hazards.

**Figure 1:**
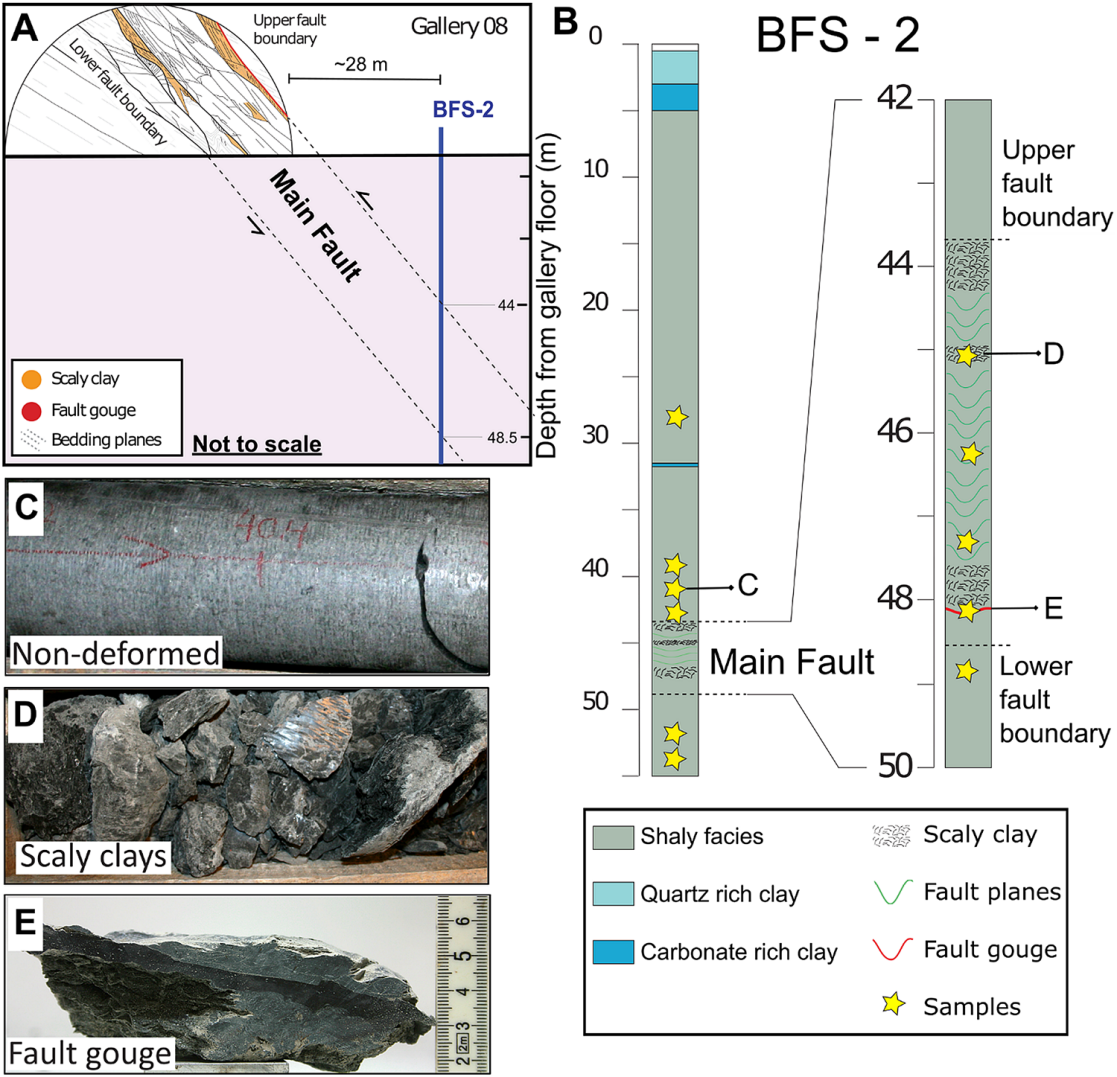
(**a**) Schema of the Main Fault intersecting Gallery 08 at the Mont Terri Laboratory. Schematic location (not to scale) of the boreholes BFS-2 intersecting the Main Fault at a depth of ~ 44 m from the gallery floor. We have modified the figure after Nussbaum et al. (2011) and Kneuker et al. (2017). (**b**). Borehole BFS-2. Lithology and structures of BFS-2 are based on detailed observations of the core. Samples discussed in this study are from borehole BFS-2 and are indicated by a star symbol in the figure. Examples of (**C**) intact (**D**) scaly clays, and (**E**) fault gouge recovered from borehole BFS-2. The position of the samples C, D, and E are indicated in figure B. Figure E from Orellana et al*.* (2018b).

At a depth of about 300 m, the MTL is intersected by faults of different scales. The major structure is a 1.0 to 4.2 m thick thrust fault dipping 50°–60° SSE named “Main Fault” (MF)^17,18^ (Fig. 1a). As part of this study, the borehole BFS-2 (Fig. 1b) was cored intersecting part of intact rock (Fig. 1c) and the Main Fault at a depth of about ~ 44 m. Within the MF, we recognize two main structural elements: a complex array of scaly clays (Fig. 1d) and a discrete fault gouge (Fig. 1e).

The intact OPA is a stiff and overconsolidated shale that shows visible bedding due to clay particle alignment^20–22^. The presence of bedding planes causes an anisotropic hydro-mechanical response of the rock, resulting in a strong transversely isotropic behaviour^23,24^. The intact OPA is characterized by good fracture sealing properties^19,25,26^ and low permeability, i.e., ~ 10^–19^ to 10^–21^ m^2,27–31^. These authors have also shown a consensus regarding permeability bedding dependency, where permeability parallel to the bedding ($k_{//}$) is higher than perpendicular to the bedding ($k_{ \bot }$), i.e., $k_{//} >$ $k_{ \bot }$. Previous mineralogical analyses have revealed that intact samples of OPA have, on average, ~ 55 to 60% of phyllosilicates, ~ 15 to 20% of calcite, and ~ 13 to 17% of quartz^32,33^.

Depending upon the method (e.g. Mercury Intrusion Porosimeter and SEM-image analysis technique, but also sample preparation: oven dry, liquid nitrogen, or wet samples), the porosity of the intact OPA usually ranges from ~ 12 to ~ 20%^21,34–38^. Nanopores, intragranular pores (e.g., framboid pyrites) and clusters of micro-cracks^34,39^ constitute the pore structure. The pore structure is preferentially oriented parallel to the bedding planes and can be considered as fully connected at the nanometer scale by pore throats smaller than 10 nm^34,39,40^. Following the work of Yu et al. (2017), more than 70% of the connected porous network is constituted by pores having diameters between 2 and 50 nm with an average pore diameter size of 13 nm.

The scaly clays in the OPA formation are zones of high shear strain characterized by complex arrangements of anastomosing slickensides enclosing lentil-shape inclusions of intact rock. While some calcite veins are recognisable in the scaly clays samples^41–45^, previous descriptions noted that scaly clays samples presents similar bulk mineralogy when compared to the host rock^31,43^.

**Figure 2:**
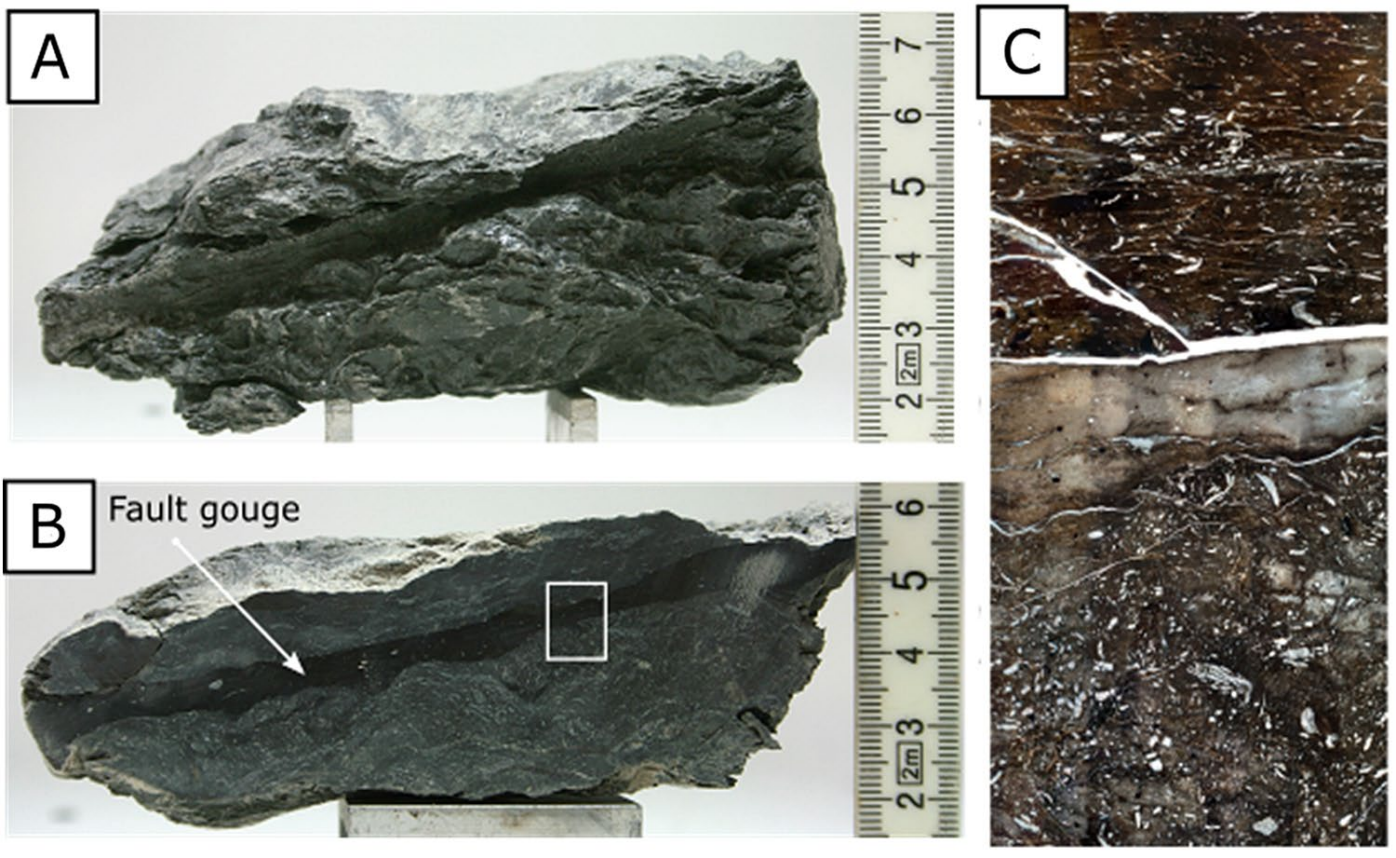
(**a**). A fault gouge sample collected from borehole BFS-2 at the Mont Terri Laboratory. (**b**). OPA Fault gouge is characterised as a 1 cm thick and dark layer, surrounded by scale clay texture. Inset (white rectangle) corresponds to figure C. Figure B from Orellana et al*.* (2018b) (**c**) Thin section image assemblage of the fault gouge sample showing the contact between the fault gouge and the surrounding scaly clay texture. As observed in the figure, the fault gouge is characterized by a strong reduction in the calcite content and small grain size, compared to the surrounding host rock. Image is in cross-polarized light.

The fault gouge in the OPA formation corresponds to a spatially discrete mm-thick gouge (Fig. 2). Earlier studies on its microstructure unveil sub-horizontal interconnected shear planes, the comminution of minerals (e.g. quartz, calcite and pyrite framboid complexes), sub-rounded grain minerals, and a strong reduction in the calcite content^41,43,45,46^. The absence of calcite has been related to the dissolution of calcite minerals by reactive fluids flowing through the fault gouges during tectonic activity^47^.

Few studies have focused on the transport properties of the MF. Based on the statistical analysis of pore size distribution on SEM images, the fault gouge has been described as a very low-porosity fault-rock (< 2%)^43,46^ compared to the surrounding intact material. Laboratory gas-permeability tests (argon) indicated that permeability magnitudes are similar in both the Main Fault and the host rock^31^. Finally, recent *in-situ* tests have revealed contradictory results. Permeability values of the surrounding rock and the Main Fault have been estimated around ~ $1 \times 10^{ - 20}$ m^2^ to ~ $1 \times 10^{ - 21}$ m^2^ with no significant difference^48,49^. A second study, however, has measured two orders of magnitude higher permeability values around ~ 10^–18^ to 10^–19^ m^2^ for the Main Fault with respect to the surrounding host rock^50^. Further, discrepancies between laboratory and field permeability and hydraulic conductivity measurements have been reported based on the inherent rock massif heterogeneity, sampling, testing procedure and fluid chemistry^31^. Thus, much uncertainty still exists about the transport properties of the MF and how they might impact the barrier condition of the Opalinus Clay formation.

## Methods

At the Mont Terri Laboratory, we selected a group of samples from the borehole BFS-2 (Fig. 1B) to perform porosity, permeability, microstructural and mineralogical analysis to assess the pore structure of the OPA fault gouge. Determining transport properties in shales has been always challenging. Samples that are not well stored or handled can be affected by non-controlled dehydration processes or mechanical stress unloading^38,51^. To minimize uncertainties due to possible artifacts, we have carefully followed standard procedures both on-site and in the laboratory^37,52^. Thus, we have ensured (as much as it was possible) the mechanical integrity and natural humidity of the natural samples. On-site treatment of samples includes reducing contact times of the fresh cores with the atmosphere, wrapping the cores in PVC bags, and then, vacuum-packing aluminum barrier foils. Once drill core samples were at the laboratory, the fresh cores were stored, while vacuum-sealed, in humidity-controlled room and carefully unpacked just before testing. Samples were taken from the inner part of the drill cores by hand or by dry sawing (when necessary) to avoid desaturation and chemical contamination.

### Sample composition and microstructures

To determine the bulk mineral composition (% weight) of our samples, we have carried out X-ray diffraction (XRD) analysis at the University of Lausanne (UNIL). The sample preparation has followed the procedure described by Kübler^53^ and Adatte et al.^54^. To get insights into the porosity microstructure, we have acquired a set of nanoscale images of the OPA fault gouge using a Zeiss Nvision 40 FIB-SEM microscopy combined with the Zeiss Atlas 5^55^ software at the Scientific Centre of Optical and Electron Microscopy (ScopeM) of ETH Zürich. Before imaging, we have dried the sample using a laboratory glass vacuum desiccator at room temperature until a constant weight was achieved. Then, we have epoxied the sample, and we have cut a cross-section perpendicular to the shear direction. Before FIB-SEM nano-tomography imaging process initiates, we have selected a region of interest (ROI) (~ 15 × 15 µm × 15 µm) using the Zeiss Atlas software. Then, an initial trench was milled at an accelerating voltage of 30 kV and beam current of 10 nA in the front part of the ROI, creating a flat surface perpendicular to the sample surface. In other words, the observed surface was then parallel to shear direction and perpendicular to the shear plane. This cross section was then polished with the FIB operating at a same accelerating voltage but using a beam current of 1.5 nA. The nanotomography was then performed by progressively sputtering thin layers of material out of the sample (~ 150 nm) with the FIB, followed by imaging with the SEM. For more details on the XRD and FIB-SEM methodology, please refer to the supporting information.

### Physical properties: porosity structure and grain density

Because of the complexity of determining porosity in shales, we have used 60 samples (seven fault gouge, eight scaly clays and 45 host rock samples) and two different fluid displacement methods on the same group of samples (< 2 cm^3^ in size): The Helium Pycnometry technique and a fluid displacement method using paraffin immersion. Since the Helium Pycnometry technique uses helium gas, the technique allows the measurements of very small pores of less than 0.5 nm in diameter. We have not used an oven-dried technique to remove pore water to avoid any additional damage to the pore structure of the samples.

We describe here our experimental protocol. First, we have measured the weight of the saturated samples $W_{sat}$. After, we have measured the weight of the saturated samples immersed in paraffin $W_{wet}$. Instead of water, we immersed the samples into paraffin thus preventing the mechanical damage of the samples because of swelling due to water absorption. The weight of the paraffin displaced is $W_{sub} = W_{sat} - W_{wet}$. Considering the value of the density of paraffin is equal to $\rho_{p} = 0.7895$ g/cm^3^, we have calculated the bulk volume of the samples $V_{b} = W_{sub} /\rho_{p}$.

Secondly, we have placed the samples into a glass vacuum desiccator until a constant weight was achieved. Once the weight was stable (~ 7 days), we have measured the dry weight $W_{d}$. The average water loss of the samples was ~ 6%. In the meantime, to obtain the HP porosity ($\emptyset_{He} )$, we have used a Micromeritics Accupyc II 1340 equipment on the same group of samples to compute the skeleton volume $V_{skel}$ of each sample. The HP porosity ($\emptyset_{HP} )$ has been calculated as $\emptyset_{HP} = (V_{b} - V_{skel} )/V_{b}$.

Later, we have crushed the samples and we have measured their final weight $W_{d}^{\prime }$ (to account for powder losses). Then, we have measured the volume of solids $V_{s}$ and deduced the grain density ρ*g* using the same Micromeritics Accupyc II 1340 apparatus. Finally, we have calculated the values of FD-porosity as $\emptyset_{FD} = (V_{b} - V_{s} )/V_{b}$.

We have measured pore throat sizes and the pore size distribution (PSD) by mercury intrusion porosimetry (MIP) at the Laboratory of Construction Materials at EPFL. The MIP is a useful technique that allows the description of pore throat sizes^56–58^. However, because it has limitations, such as the lack of direct access to the pore volume^59^, we have restricted our discussion of the MIP results mostly to a qualitative interpretation. Thus, we have not computed porosity from this technique. The throat size distribution might not exactly match the pore size distribution (PSD), but can be considered as a first-order approximation^57,58^.

The MIP procedure is as follows. First, we have dehydrated the sample (< 1 cm^3^) for about 24 h using a freeze-drying technique^60^. Then, we have placed the sample in a sample-cell holder who was filled with mercury at low pressure (up to 400 kPa) to penetrate the largest pores in the sample. After, we have emplaced the sample holder in a high-pressure system where pressures of up to 440 MPa were applied. Both the isostatic pressure $P$ and the intruded volume of mercury $V_{m}$ were recorded continuously. We have processed the MIP data using the Washburn equation:

1 $$\Delta P = - \frac{4\gamma \cdot \cos \theta }{d}$$

where $P$ is pressure, $\gamma$ is the surface tension of mercury, and $\theta$ the contact angle between the solid and mercury. We used $\gamma = 0.486 \;N/m$ and $\theta =$ 142°^60^. Assuming non-intersecting cylindrical pores geometries, we were able to determine the diameter of the pore throat $d$.

As indicated before, we carefully handled the samples during collection and testing. However, some possible mechanical damages (e.g. clay shrinkage and so micro-cracks) might have occurred since drying processes were required when measuring porosity via HP, FD, and MIP techniques^35,51^.

While permeability testing on intact samples from the host rock and the Main Fault is possible, as shown by several authors, accurate permeability measurements of the OPA fault gouge and scaly clays is almost impossible. Because of their geometry and mechanical properties^41,43^, it is not possible to core a well-defined sample of scaly clays or fault gouge suitable for testing (Figs. 1, 2).

## Results

### Samples composition

From the X-ray diffraction (XRD) measurements (for detailed information on the XRD procedure, please refer to the Supplementary Material), we have identified phyllosilicates, quartz, calcite, and pyrite as the main constituents of the samples (Table 1). The mineral composition of the Opalinus Clay host rock samples consists of phyllosilicates (~ 51%), quartz (~ 23%), calcite (~ 14%), and pyrite (~ 1.4%). The group phyllosilicates correspond to the sum of illite–smectite, mica, chlorite, and kaolinite.

**Table 1:** Bulk (% weight) mineralogical composition of Opalinus Clay samples. We present results as X ± S, where X is the mean and S the standard deviation; “n” corresponds to the number of samples.

Mineralogy (wt%)	Fault gouge (n = 4)	Scaly Clays (n = 3)	Ca-rich veins Scaly clays (n = 4)	Intact (n = 6)
Quartz	26.5 ± 1.4	20.6 ± 0.9	22.7 ± 1.7	23.0 ± 0.9
Feldspath-K	3.3 ± 0.2	2.2 ± 0.2	2.9 ± 0.4	2.3 ± 0.4
Plagioclase-Na	3.0 ± 0.7	3.8 ± 2.5	2.5 ± 0.5	2.3 ± 0.2
Calcite	2.1 ± 0.4	17.5 ± 4.6	19.9 ± 3.0	14.2 ± 1.3
Dolomite	0.7 ± 0.4	1.4 ± 0.4	0.0 ± 0.0	1.3 ± 0.3
Pyrite	2.5 ± 0.3	1.4 ± 0.5	1.1 ± 0.3	1.4 ± 0.3
Goethite	1.4 ± 1.4	1.4 ± 1.0	1.8 ± 1.1	2.1 ± 0.3
Illite–Smectite	11.7 ± 5.5	3.0 ± 0.9	2.7 ± 0.6	4.0 ± 2.3
Mica	11.8 ± 4.9	8.8 ± 1.4	7.0 ± 1.3	10.3 ± 3.6
Chlorite	9.9 ± 4.3	9.3 ± 1.0	10.5 ± 3.3	8.5 ± 2.4
Kaolinite	23.4 ± 6.2	28.2 ± 2.1	26.9 ± 1.2	28.0 ± 2.9
Others	3.6	2.4	1.9	2.7

**Figure 3:**
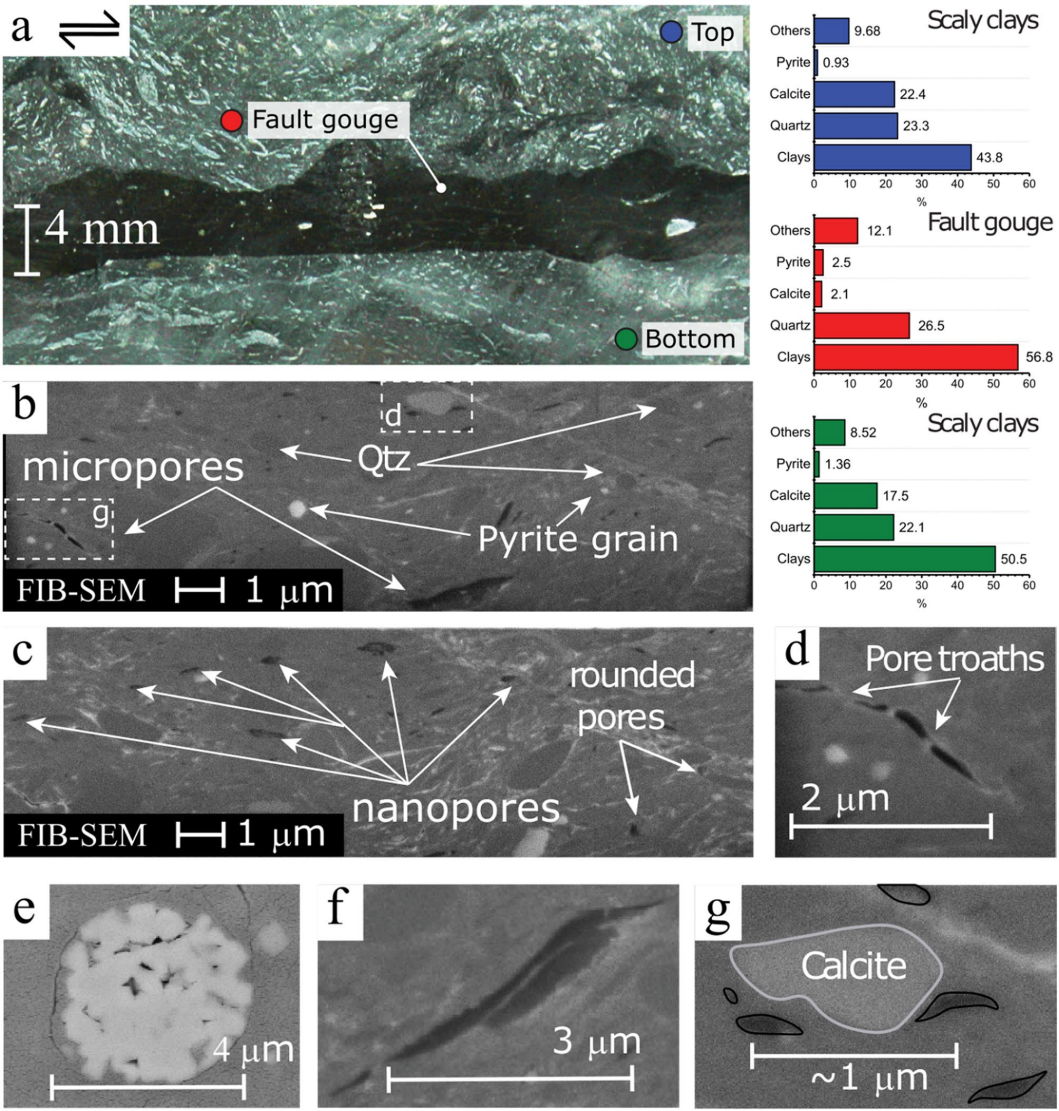
Microstructures of the fault gouge. (**a**) A cm-scale photograph of the fault gouge (black) recovered from borehole BFS-2. The thickness of the gouge varies from 3 to 10 mm, and it is surrounded by calcite-enriched scaly clays. On the right, three graphs showing the different mineralogical composition of the gouge and its surroundings. (**b**) Selected FIB-SEM image of the fault gouge showing micropores. White boxes show the position of figures (**d**) and (**g**). The image also shows rounded quartz minerals, isolated pyrite grains and rare presence of calcite. (**c**) Selected FIB-SEM image of the fault gouge showing sub-rounded and sub-angular nanopores aligned in the direction of shearing. Isolated rounded pores are also present (**d**) Thin and elongated micropores connected in 3D by pore throats. (**e**) SEM images of framboid pyrite with porosity in the single pyrite grains. (**f**) FIB-SEM image of angular or jagged micropores connected in 3D by pore throats. (**g**) Nanopores possibly connected by pore throats situated near a calcite grain boundary i.e., pore-grain bridge complex. Images (**E**) and (**F**) were obtained from other sections of the sample.

We observe a higher amount of calcite content (~ 17%) in the scaly clays and the calcite-rich scaly clays near the fault gouge (> 17%) (Fig. 3a). Here, we refer to calcite-enriched scaly clay to the scaly clays samples that are 5% higher in calcite content (thanks to calcite veins) than the most common scaly clay samples which are collected far from the fault gouge.

Based on XRD measurements, we recognize a proportion of calcite minerals significantly smaller (~ 2%) in the fault gouge compared to the intact and scaly clay rocks (~ 14–20%). We also observe different phyllosilicates including illite–smectite, kaolinite, mica, and chlorite. Among them, kaolinite is the dominant constituent in all samples. Further, there is a significantly higher proportion of illite–smectite in the gouge compared with the other samples, and a higher compositional variability within the gouge (high standard deviations in the phyllosilicate fraction) compared to the other samples.

### The microstructure of the fault gouge

The fault gouge is a black layer of about 3 to 10 mm thickness (Figs. 2, 3a). The fault gouge contains clays minerals oriented parallel to shear direction, sub-rounded quartz, and isolated pyrites minerals with angular edges (Fig. 3b,c). In agreement with previous observations^43^, the fault gouge is characterized by (i) a strong reduction in the calcite content and small grain size, compared to the surrounding host rock (Fig. 2c) and (ii) an inner structure defined by an S-C fabric in the gouge with dextral shear sense (see Supplementary Material, Fig. S3).

The fault gouge is surrounded by calcite-enriched scaly clay and calcite veins as revealed by the mineralogical composition (Fig. 3a). Here, we refer to calcite-enriched scaly clay to the group of scaly clays samples that present a higher calcite content thanks to calcite veins presence (5% higher in average). The top fault gouge-scaly boundary (blue circle in Fig. 3a) appears as a wavy or non-regular surface, and the associated calcite-enriched scaly clay reveals a complex pattern as suggested by the variable orientation of the calcite minerals. In contrast, the bottom fault gouge-scaly boundary is fairly regular or flat (green circle in Fig. 3a). The bottom scaly clays that appear aligned parallel to the fault gouge are dominated by a laminar (sheet-like) flow, as highlighted by the orientation of calcite minerals. Such type of alignment can be also observed or enhance due to fault-perpendicular compression mechanisms.

Following the FIB-SEM observations, we recognize an apparent low-porosity matrix, with nano and micro-pores with a radius of about < 100 nm and lengths < 3 µm, respectively, that appear aligned parallel to the foliation defined by the phyllosilicates and are connected in 3D (more images in Supplementary Material). While nanopores are of rounded and sub-rounded shapes of typically < 30 nm (Fig. 3b,c), micropores show thin, elongated and sub-angular shapes and they are connected in 3D by pore throats that follow the foliation defined by the phyllosilicates (Fig. 3d,f). Also, we observe intragranular nanopores in framboid pyrites (Fig. 3e) and intergranular pores of sub-rounded shapes (diameter < 30 nm) forming a pore-grain bridge complex i.e., nanopores possibly connected by pore throats situated near a calcite grain boundary (Fig. 3g).

### Porosity of intact rock and the internal structures of the OPA fault core

**Figure 4:**
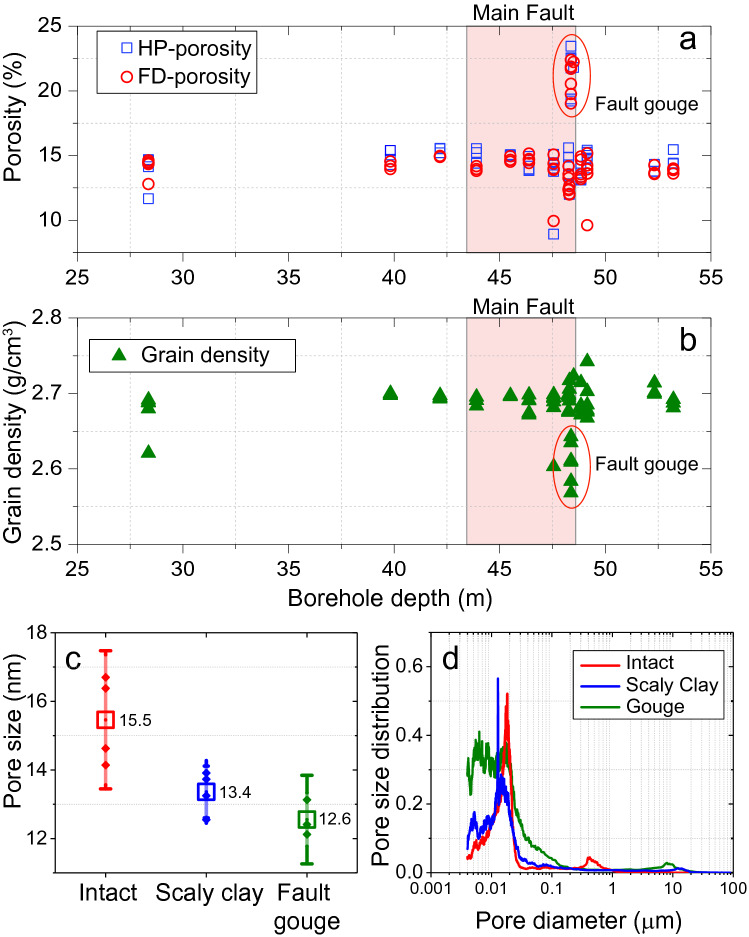
(**a**) HP and FD porosity. (**b**) grain density. (**c**) MIP pore throat average sizes, and (**d**) PSD measurements of the intact and fault-related rocks of borehole BFS2. The MF is highlighted in red in (**b**) and (**c**). Values in (**c**) correspond to pore throat average sizes measured in each MIP test.

The HP-porosity (Fig. 4a, Table 2) is 14.2 ± 1.1% (average ± standard deviation) for intact samples, 13.4 ± 1.2% for scaly clays and 21.4 ± 1.5% for the fault gouge. Similarly, FD-porosity (Fig. 4a, Table 2) shows values of 13.9 ± 1.1% for intact, 12.7 ± 0.7% for scaly clays, and 20.9 ± 1.2% for fault gouge samples. The grain density results (Fig. 4b, Table 3) indicate values of 2.69 ± 0.02 g/cm^3^ for the intact, 2.69 ± 0.01 g/cm^3^ for scaly clays, and 2.62 ± 0.05 g/cm^3^ for the fault gouge samples. For more details, please refer to the supporting information.

**Table 2:** Porosity results of intact, scaly clay and fault gouge Opalinus Clay samples.

Measurement	Helium pycnometer porosity ${\varnothing }_{HP}$ (%)			FD—porosity ${\varnothing }_{FD}$ (%)		
Sample	Intact	Gouge	Scaly	Intact	Gouge	Scaly
Mean	14.2%	21.4%	13.4%	13.9%	20.9%	12.7%
Max	15.4%	23.3%	15.5%	15.0%	22.3%	14.0%
Min	8.9%	19.2%	11.9%	9.5%	18.9%	11.9%
St. Deviation	1.1%	1.5%	1.2%	1.1%	1.2%	0.7%

**Table 3:** Grain density results of intact, scaly clay and fault gouge Opalinus Clay samples.

Measurement	Grain density [g/cm^3^]		
Sample	Intact	Gouge	Scaly
Mean	2.69	2.62	2.69
Max	2.74	2.72	2.72
Min	2.60	2.57	2.68
St. Deviation	0.02	0.05	0.01

The MIP measurements indicate an average pore throat diameter of 15.5 ± 1.1 nm for intact samples, 13.4 ± 0.5 nm for scaly clays and 12.6 ± 0.4 nm for fault gouge (Fig. 4c). The PSD curves show that dominant pore throat size ranges are between ~ 4 and ~ 20 nm for all the tested samples (Fig. 4d). However, the PSD curves also show that pore throats of less than ~ 10 nm exert an important control on the pore structure of fault gouges. In contrast, the dominant spectrum of pore throat diameter is higher and around ~ 12 and ~ 20 nm for scaly clays and intact rocks, respectively.

## Discussion

### Comparison of results with previous studies

Previous studies have mostly focused on the microstructural characterization, and the mechanical and transport properties of intact samples cored from the host rock of OPA formation^21,22,61,62^. Conversely, less attention has been paid to its faulted zones and the role of the internal fault core structure^17,31,43,45,47–49,63–65^. Thus, in this section, we discuss our results by comparing them with earlier studies of intact samples.

#### Sample composition

For the intact samples, our results indicate a dominant proportion of phyllosilicates (~ 51%) followed by quartz (~23%) and calcite (~14%) as main constitutive minerals (Table 1). These results are consistent with data obtained in earlier studies^32,33^. Our XRD measurements show (1) an important reduction in calcite (~ 2%) in the fault gouge with respect to intact OPA (~14%), and (2) calcite-enrichment of scaly clays (~22–18%) due to calcite veins in the immediate surroundings of the fault gouge. Previous microstructural observations^17,43,46^ have qualitatively described a similar lack of calcite within the mineralogical composition of the fault gouge.

#### Porosity and grain density

We have collected and tested 60 OPA samples to ensure the reproducibility of our porosity (Table 2) and grain density (Table 3) measurements at different positions from the borehole BFS-2 (Fig. 1b), including 45 samples of intact OPA, 8 of scaly clays and 7 of fault gouges. Prior studies have noted the importance of the internal fault core in the Main Fault and their implications to nuclear waste storage and caprock integrity. Yet, a main disadvantage is the absence or lack of samples from the Main Fault due to their sampling costs. peculiarity, and elusiveness. While scaly clays and fault gouge samples of the OPA formation (Figs. 1, 2) in this study are not numerous compared to intact OPA samples, this work presents a unique dataset of their mineralogical and physical properties.

For intact samples, our porosity data agree with those of various authors who have reported values of around 12 to 15%^22,30,34–36,39,40^. For scaly clays, our porosity results appear consistent with previous ones based on the SEM-image analysis of zones near slickensides surfaces (ϕ ~ 19%)^43^. For the fault gouges, values of porosity less than 2% were recorded from SEM-based images^43,44,46^. In our study, the fault gouge average porosity of ~ 21% was measured using fluid displacements methods. These discrepancies could be related to the high spatial variability of the microstructural and transport properties within the whole formation, but also to different technical capabilities. Indeed Fig. 3c shows that an important portion of the pores throat sizes is below 10 nm, thus necessarily being out of the measurement resolution space of the SEM-image technique^46,66^.

Finally, small pore throat diameters of about ~ 15 nm and a grain density of 2.69 g/cm^3^ also characterize the intact Opalinus Clay samples. Similarly, earlier results have reported comparable values of ~ 13 nm and 2.74 g/cm^3^ for pore throat diameter and grain density, respectively^30^.

### Mineralogical composition of the OPA fault gouge

A closer look at the fault gouge and the fault gouge—scaly clay boundary (Fig. 3a) reveals an assorted distribution of calcite content, calcite grain sizes, and shapes. As described before, the OPA fault gouge microstructure displays a notorious absence of calcite (Table 1 and Fig. 3a), which is consistent with the reduction in grain density (Fig. 4b). In Fig. 3a, the bottom fault gouge-scaly clay boundary predominantly presents elongated calcite veins and small calcite minerals mostly oriented parallel to the fault gouge. Further, the calcite content of the bottom fault gouge-scaly clay is lower than in the top fault gouge-scaly clay boundary (17.5 vs. 22.4%) but higher than in the fault gouge (~ 2%) and the intact samples (~ 14%). On the other hand, the top scaly-gouge boundary evidences a higher concentration of calcite grains and calcite veins distributed in an unclear spatial pattern (Fig. 3a).

An explanation for the absence of calcite and the inhomogeneous distribution of calcite grains and veins in the surroundings of the fault gouge is that carbonate-reactive fluids have played an important role at different stages of the tectonic activity of the Main Fault as explained by Clauer et al. (2017). As calcite minerals can deform by fracturing at low-grade conditions^67^ (i.e., increasing the area of contact), reactive fluids flowing throughout the fault gouge could have easily dissolved and transported the comminuted calcite outside the fault gouge limits, where they recrystallized. Also, the fact that calcite crystallized in the surroundings of the fault gouge can be evidence that, at the time of calcite precipitation, the porosity in the fault gouge surroundings was higher.

This process can only occur if the fault gouge was more permeable, thus acting as a preferential path for fluid flow. In our study, we have shown that the fault gouge has a relatively higher porosity with respect to the surrounding rock mass (21% vs. 14%), thus pairing the field evidence and supporting this hypothesis. Because water is present, pressure solution may have also played an important deformation role^67^. However, it is difficult to differentiate its relative contribution to the total calcite dissolution process.

### New insights into the Main Fault-related fluid-flow structure

Porosity and permeability are related transport parameters that are vastly used to characterize the capacity for fluids circulation in fault zones. Unfortunately, we have strong limitations to collect and directly test permeability on OPA fault gouges and scaly clays samples. While direct measurements are the most accurate source of information, measuring the permeability of the OPA fault gouge and scaly clays is almost impossible due to their geometry, mechanical properties, and current testing apparatus.

Following the previous discussion, in this section, we attempt to provide a conceptual model of the Main Fault-related fluid-flow based mainly on our porosity data, porosity topology, and different permeability models following the approaches of different authors. There are no obvious nor direct porosity–permeability relationships that can allow us to estimate the permeability of the mentioned fault rocks. Yet, their fluid permeability might be assessed with some models we describe below. By doing so, these models allow us to provide new insights into the fluid flow structure of the Main Fault at MTL, and in the role of the internal fault core structure of the Opalinus Clay formation. To evaluate the accuracy of these models, we first compared them with data from permeability tests we have performed on intact OPA samples cored parallel to bedding. These tests have shown permeability decreases from ~ 3 × 10^–19^ to ~ 4 × 10^–20^ m^2^ when the effective pressure increases from 2 to 12 MPa respectively (see Supplementary Material for details, Figs. S1, S2). We focus on the capacity for fluid flow parallel to the direction of shearing since samples perpendicular to the shear direction have lower permeability, as shown by earlier works^27,29,31^.

#### The role of the internal fault core based on tube and crack permeability models

We follow Guéguen & Dienes^68^ and Guéguen & Palciauskas^69^ method for estimating permeability. Thus, we simplify the permeability ($k$) estimation by assuming that the pore network of the OPA fault rocks is either characterized by a network of interconnected crack-like pores (i.e., interconnect sheets) or interconnected tube- and sphere-like pores. These classical models represent two end members of possible pore structures.

If we assume that the porosity can be represented by a homogenous and isotropic distribution of tubes, then its permeability $k_{tubes}$ can be estimated as $k_{tubes} \cong \frac{{r^{2} }}{8}\emptyset$. where $r$ is the tube radii and $\emptyset$ is the porosity. Following the MIP and porosity results (Table 2), we use the average values of porosity and throat size to estimate the permeability of each group of samples. Thus, the tube model provides values of permeability of $\sim1 \times 10^{ - 18}$ m^2^ for the intact rock, $\sim7{ } \times { }10^{ - 19}$ m^2^ for the scaly clays, and $\sim1{ } \times { }10^{ - 18}$ m^2^ for the fault gouge. The measured permeability for intact OPA is in the order of $\sim2.5{ } \times { }10^{ - 19}$ m^2^ at 5 MPa confining stress, i.e., one order of magnitude lower than the estimated value from the tube/sphere model. The latter suggests that the characteristic pore size for fluid transport should be smaller than 15.5 nm to match modelled and estimated values of permeability. Indeed, it requires pore diameters of less than 7 nm. Further, it appears least consistent with the dominant spectrum of pore throat diameter of ~ 12 and ~ 20 nm (Fig. 4d) and with the earlier description of a connected porous network of mesoporous (13 nm mean size) within the intact OPA^30^. However, we have shown that the fault gouge contains a large portion of pore throats of less than 10 nm (Fig. 4d) and nanopores of sub-rounded shapes (Fig. 3) matching some of the geometrical characteristics of this pore network model.

Alternatively, if the porosity is the result of straight interconnected crack-like pores, its permeability $k_{cracks}$ can be estimated by $k_{cracks} \cong \frac{{w^{2} }}{3}\emptyset$, where $2w$ corresponds to the aperture of the cracks. We assume $2w$ to be equivalent to pore throat size entry. Using the average value of porosity and pore throats sizes for each type of rock, the crack model provides values of permeability of $\sim3 \times 10^{ - 20}$ for the intact rock, $\sim2{ } \times { }10^{ - 20}$ m^2^ for the scaly clays, and $\sim3{ } \times { }10^{ - 20}$ m^2^ for the fault gouge. In this scenario, calculated permeability is one order of magnitude lower than measured permeability ($\sim1{ } \times { }10^{ - 19}$ m^2^) at 5 MPa confining stress. The last point suggests a control by interconnected sheet or crack-like pores^70,71^, which in turn is consistent with pores more or less oriented along clay particles interfaces in the intact rock^30,34,39,40^. The preferential orientation of the pore network, including sheet-like pores is also observed in the FIB-SEM images of fault gouge (Fig. 3). Thus, if we extrapolate the permeability dependence of the intact rock to the fault gouge, we might infer that the pore structure of the fault gouge responds to effective pressure variations in the same way as cracklike pore networks.

Obviously, none of these two end-member models can represent the complex nature of porous shales. Indeed, we have seen that none of these two models can explain the permeability of the intact rock by itself. Thus, when characterizing the pore network of the intact and the OPA fault rocks, we should expect a combination of both, where a heterogeneous network of crack-like and tubes/sphere like pores coexist.

#### Estimation of permeability based on hydraulic radius models

We have tested the classical semi-empirical relationship of Kozeny–Cartman (KC)^72,73^. The KC equation is based on a geometrical approach where the pores are modelled as a group of capillary tubes. Permeability from the KC can be calculated as ${\varvec{k}} = \frac{{{\varvec{d}}^{2} \emptyset^{3} }}{{180\left( {1 - \emptyset^{2} } \right)}}$ where ${\varvec{d}}$ corresponds to the mean pore diameter. The KC model predicts values of permeability of $\sim4 \times 10^{ - 21}$ m^2^ for the intact rock, $\sim2 \times 10^{ - 21}$ m^2^ for the scaly clays, and $\sim9 \times 10^{ - 21}$ m^2^ for the fault gouge. These values are 2 orders of magnitudes smaller than the permeability values measured on intact OPA presented (see Supplementary Material for details, Figs. S1, S2).

#### Estimation of permeability based on empirical models

Finally, we have used two empirical relationships, one of them constructed for shales that include the clay content as a parameter, and the second specifically for intact OPA. Based on a large dataset of shale properties. Yang and Aplin (2010) have introduced a permeability–porosity ($\emptyset$) of the form $\ln ({\varvec{k}}[) = \user2{ a}\left( {\varvec{\delta}} \right) + {\varvec{b}}\left( {\varvec{\delta}} \right) \cdot \frac{\emptyset }{1 - \emptyset } + {\varvec{c}}\left( {\varvec{\delta}} \right) \cdot \left( {\frac{\emptyset }{1 - \emptyset }} \right)^{0.5}$, where ${\varvec{a}}$, ${\varvec{b}}$, and ${\varvec{c}}$ are constants that depend on the clay content (for details, see Supplementary material). The Yang and Aplin (2010) model predicts values of permeability of $\sim9 \times 10^{ - 21}$ m^2^ for the intact rock, $\sim8 \times 10^{ - 21}$ m^2^ for the scaly clays, and $\sim3 \times 10^{ - 20}$ m^2^ for the fault gouge. To compute these values, we have used a clay content of 51% for intact rock, 49% for scaly clays, and 57% for fault gouge samples.

The second empirical porosity–permeability relationship is a laboratory-based model for intact OPA in the form ${\varvec{k}} = 1.05 \times 10^{ - 17} \cdot \frac{{\emptyset^{3} }}{{\left( {1 - \emptyset } \right)^{2} }}$^74^. The Muñoz et al. (2009) model predicts values of permeability of $\sim3 \times 10^{ - 20}$ m^2^ for the intact rock, $\sim2 \times 10^{ - 20}$ m^2^ for the scaly clays, and $\sim1 \times 10^{ - 19}$ m^2^ for the fault gouge. These values are not consistent with the permeability measured on intact OPA. Other empirical relationships might be found in the literature; our objective is not testing them all but rather illustrate different approaches.

#### Implications for fluid flow in OPA fault zones

We have presented empirical^74,75^, statistical (tubes/spheres and cracks, from Guéguen & Palciauskas (1994)). and hydraulic^72,73^ permeability models to account for a hypothetical model of the Main Fault fluid flow. A summary of the results is presented in Table 4.

**Table 4:** Mean experimental porosity (%) and modeled permeability (m^2^) of intact, scaly clay, and fault gouge samples. Mean values correspond to the average of all experimental data for each sample.

Sample	Mean ϕ (%)	Modeled permeability (m^2^)				
		Tubes	Crack-like pores	Kozeny–Cartman (1927)	Muñoz et al. (2009)	Yang and Aplin (2010)
Intact	14.1	1E−18	3E−20	4E−21	3E−20	9E−21
Scaly clay	13.0	7E−19	2E−20	2E−21	2E−20	8E−21
Gouge	21.2	1E−18	3E−20	9E−21	1E−19	2E−20

Obtaining an expression that establishes an accurate porosity–permeability relationship for shales is in general difficult. Moreover, this aim will be ambitious for fault-related clay-rich rocks. Although previous models can be considered simplistic and optimistic, they provide useful insights into the fluid flow distribution and locations of preferential pathways in the Main Fault. The tube and crack models indicate a null increase in the fluid permeability of the fault gouge with respect to the intact rock. Yet they reveal that the scaly clays are the less permeable among the three groups of samples. The remaining models suggest an increase of up to 3 times the magnitude of permeability of the OPA fault gouge relative to scaly clay and intact material. This permeability increase is more pronounced when the porosity–permeability model is a function of either porosity^74^ or porosity and clay content^75^. The permeability increase is smaller in the KC equation compared to these two empirical models, as it depends only on the diameter of the pores^72,73^.

Based on experimental data, different permeability models lead to different scenarios of fluid permeability. While some of them suggest there is no significant difference between permeabilities of the different fault compartments (KC equation, crack-like pores), none of these models leads to a fault gouge acting as an active barrier. In addition, the microstructural evidence of dissolution and transport of calcite around the fault gouge, as shown here and by other authors^43,46,47^, favours the scenario of the fault gouge being more permeable than the surrounding host rock. Despite the virtual permeable role of the fault gouge, both the modelled and experimental permeability values (~ 10^–18^ to ~  × 10^–20^ m^2^) suggests the OPA formation maintain its barrier condition.

Finally, three notes of caution should be considered. The first is related to the scaly clay samples used in this study. The tested scaly clay samples are less than 2 cm^3^ in size, thus they might not represent the complexities of long fractures within scaly clay fabrics as observed in the Main Fault, and therefore some larger-scale features are possibly missing^42^. Also, when the content of smectite or mixed layers of illite/smectite is high enough, self-sealing mechanisms such as clay-swelling acting on fractures can be argued as an effective mechanism against a more favorable conduit pathway for fluid flow^19,76,77^. Apparently, this is unclear in our samples. A second point to consider is that all conclusions concerning permeabilities in the fault compartments are mostly based on the estimates derived from different porosity–permeability models. Since they are not direct measurements, they should be considered carefully. However, it is important to notice that measuring the permeability of the OPA fault gouge and scaly clays is extremely complicated due to their geometry, mechanical properties, and current testing apparatus. Finally, water-bound porosity can be significant and can alter the relationship between porosity and permeability in shales. To test this hypothesis, cation exchange capacity (CEC, i.e., to test the capacity to retain cations) should be performed on OPA fault gouge samples. A detailed analysis of the contribution of water-bound porosity to the total porosity of the samples is out of the scope of this paper. Despite some limitations, this study provides new insights into the permeability structure of the Main Fault and related fault compartments at the Mont Terri Laboratory.

Following our results, a Main Fault fluid transport schema based on a higher-porosity higher-permeability fault gouge is in general agreement with previous studies of fault gouge samples collected along natural clay-bearing faults^78,79^. However, our study differs from those typically reported in classical models referring to clay-rich low-permeability fault cores^7,9,10^. If this interpretation is correct, the relative higher permeability and porosity of the OPA fault gouge compared to its rock mass surroundings, is consistent with a localized permeable conduit structure^6^. More testing is needed to evaluate this interpretation.

## Conclusions

The Opalinus Clay formation is a suitable deep geological repository as a potential host rock for nuclear waste storage in Switzerland. Because of the low permeability of intact shales, hydrological concerns in the context of nuclear waste storage have been focused on disturbances caused by mechanical and thermal effects during excavation and operation, respectively. As we have shown, faults, however, impose new challenges on the long-term operation of these repositories. Through microstructural and pore network characterization, we present an analysis of the hydrological behavior of the clay-rich fault gouge within a major fault system (MF) intersecting the Opalinus Clay (OPA) formation at the Mont Terri Laboratory (MTL), a host-rock candidate for deep nuclear waste storage in Switzerland.

Based on laboratory evidence, we have shown (1) the absence of calcite within the fault gouge suggesting that calcite might have been dissolved by a reactive fluid flowing throughout the fault gouge, (2) a re-crystallization of calcite veins and blocky grains in the surrounding of the calcite-enriched scaly clays due to precipitation of the dissolved calcite in the vicinity, (3) a high porous fault gouge (~ 21%) corresponding to a low grain density(2.62 ± 0.05 g/cm^3^) when compared to the intact rock (~ 14% of porosity and 2.69 ± 0.02 g/cm^3^ of grain density). In addition, based on semiempirical porosity–permeability relationships, we suggest a non-homogenous distribution of fluid-flow in the Main Fault as a consequence of the architecture and the related spatial variability of the physical properties. Thus, we infer, in agreement with the field and laboratory observations, that the OPA fault gouge might act as a discrete internal fault structure having the potential to act as preferential path for fluid flow. However, if this occurs, it will be only limited to a narrow (millimetres-thick), discontinuous and tortuous fluid-channel.

Because the OPA formation is a potential host rock for nuclear waste storage, the implication of the fluid-flow governing behavior is of critical importance for the society and the environment. The Opalinus Clay formation at the MTL is not meant to be the final repository. However, if fluid-flow of radionuclides occurs within a fault system array in the OPA formation, it will take place following an uneven contribution, between a more permeable but limited in volume, narrow and spatially discrete fault gouge, versus a more extensive low permeability and self-sealing argillaceous host rock. Thus, we expect the favorable barrier and hydrological properties of the Opalinus Clay formation is not affected, and the integrity of the nuclear waste repository safeguarded.

## Supplementary Information

Supplementary Information.
