# Supplementary material for: Physical characterization of fault rocks within the Opalinus Clay formation
Source: Sci Rep. 2022 Mar 14;12:4389. doi: 10.1038/s41598-022-08236-7 (PMC8921326; doi:10.1038/s41598-022-08236-7)
Supplement: Supplementary file 1 — Supplementary Information. [file 41598_2022_8236_MOESM1_ESM.docx]

*Scientific Report*

Supporting Information for

Physical characterization of fault rocks within the Opalinus Clay formation

Luis Felipe Orellana^1,5,6*^, Christophe Nussbaum^2^, Luiz Grafulha^3^, Pierre Henry^4^, Marie Violay^1^

^1^ Laboratory of Experimental Rock Mechanics, IIC-ENAC, École Polytechnique Fédérale de Lausanne, Lausanne, Switzerland.

^2^ Federal Office of Topography (Swisstopo), Wabern, Switzerland.

^3^ Scientific Centre of Optical and Electron Microscopy, ETH Zürich, Zürich, Switzerland

^4^ [Centre Européen de Recherche et d’Enseignement des Géosciences de l’Environnement](https://www.researchgate.net/institution/Aix-Marseille_Universite/department/Centre_Europeen_de_Recherche_et_dEnseignement_des_Geosciences_de_lEnvironnement), [Aix-Marseille Université](https://www.researchgate.net/institution/Aix-Marseille_Universite), France.

^5^ Now Department of Mining Engineering, FCFM - Universidad de Chile, Santiago, Chile.

^6^ Now Advanced Mining Technology Center (AMTC), FCFM – Universidad de Chile, Chile

Corresponding author: Luis Felipe Orellana ([luisfelipe.orellana@ing.uchile.cl)](mailto:luisfelipe.orellana@ing.uchile.cl))

Contents of this file:

1. A Detailed section of methods: sample composition (XRD analysis) and microstructures (FIB-SEM imaging).
2. The permeability tests method and results on intact sample cored parallel to bedding
   - **Figure S1:** Permeability setup
   - **Figure S2:** Permeability results
3. Additional FIB-SEM images of the OPA fault gouge.
   - **Figure S3:** FIB-SEM images of the OPA fault gouge
4. Summary of porosity and grain density results
   - **Table S1:** Summary of porosity and grain density results
5. The model of Yang & Aplin, (2010) based on an exponential law
6. Methods: Sample composition and microstructures

To determine the bulk mineral composition (% weight) of our samples, we have carried out X-Ray diffraction (XRD) analysis at the University of Lausanne (UNIL). The sample preparation has followed the procedure described by Kübler (1987) and Adatte et al., (1996). The procedure has included: a) the disaggregation of the rock sample and mixing with de-ionized water, b) the subtraction of the carbonate portion by the addition of HCl 10% c) the separation of the different grain size fractions (< 2µm and 2-16 µm) using the timed settling method (Stokes law), d) the application of ethylene-glycol onto clays already placed on glass-slide, and finally, e) the recognition of characteristic XRD peaks of each clay mineral.

To get insights into the pore structure microstructure, we have acquired a set of nanoscale images of the OPA fault gouge using a Zeiss Nvision 40 FIB-SEM microscopy combined with the Zeiss Atlas software at the Scientific Centre of Optical and Electron Microscopy (ScopeM) of ETH Zürich. Before imaging, we have dried the sample using a laboratory glass vacuum desiccator at room temperature until a constant weight was achieved. Then, we have epoxied the sample, and we have cut a cross-section perpendicular to the shear direction. We have polished this surface using a broad ion beam (BIB) equipment (IM4000 Hitachi). After, we have coated the sample with a 30 nm film of 80/20 - Platinum/Palladium alloy using a CCU-010 sputter coater equipment. Before FIB-SEM nano-tomography imaging process initiates, we have selected a region of interest (ROI) (~15 x 15 µm x 15 µm) using the Zeiss Atlas software. The selection of a ROI dimension of ~ 15x15x15 is based on: 1), it allows the detailed imaging of the fine porosity, mineralogy and fractures with a good level of detail (i.e voxel size of 10 nm), and also 2) data acquisition is a relatively short time (<24 h), bit compromising the stability of data acquisition (charging/drifting) in a complex material such as clays.

The ROI was then covered with a 2 µm layer of platinum for protection. Fiducial marks were delineated for auto-drift correction and auto-focus, and a layer of carbon is deposited to enhance image contrast. Both layers were deposited using the FIB operated at 30 kV and 150 pA beam current. After that, an initial trench was milled at an accelerating voltage of 30 kV and beam current of 10 nA in the front part of the ROI, creating a flat surface perpendicular to the sample surface. This cross section is then polished with the FIB operating at a same accelerating voltage but using a beam current of 1.5 nA. The nanotomography was then performed by progressively sputtering thin layers of material out of the sample (~150 nm) with the FIB, followed by imaging with the SEM. During image acquisition, accelerating voltage and beam current were, respectively, 30 kV and 1.5 nA for the FIB, and 3 kV and ~80 pA for the SEM.

1. Permeability tests method and results on intact sample cored parallel to bedding

To measure the permeability of the OPA fault gouge and scaly clays is very challenging. Because of their geometry and mechanical properties ^4,5^, it is not possible to core a well-defined sample suitable for testing. Hence, as fluid-flow through pores parallel to the bedding direction is the most favorable scenario ^6^, we have decided to measure the permeability of intact OPA samples cored parallel to bedding. This data is then used to model the permeability of fault-related rocks based on porosity-permeability relationships (see main text).


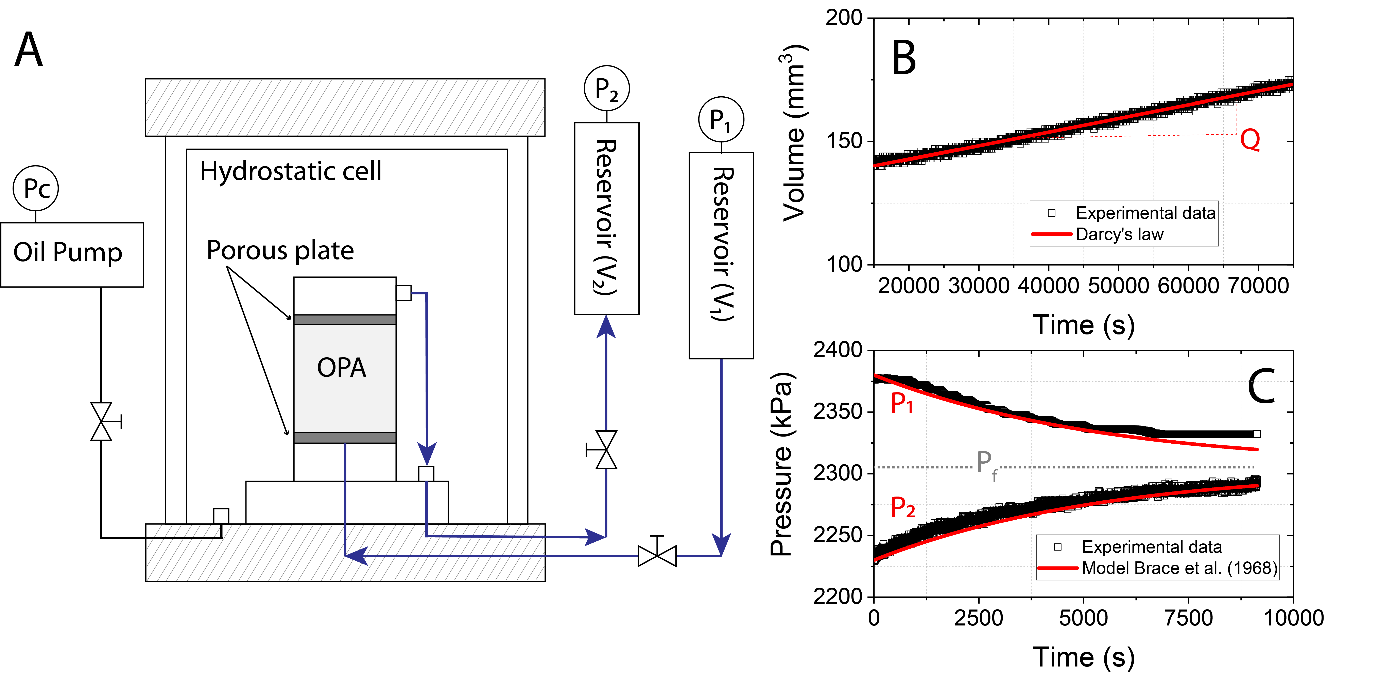


**Figure S1:** Permeability measurements. (a) The setup used in this study. (b) Calculation of Q (mm^3^/s) following Darcy’s law for test at 4 MPa effective confining stress. (c) Following the transient step method of Brace et al. (1968), the pressure $P_{1}$ and $P_{2}$ evolved exponentially over time until they approached a final pressure $P_{f}$. The figure shows an example of measurement at 5 MPa effective confining stress.

We carried out permeability measurements in a hydrostatic cell at different effective confining pressures (2 to 12 MPa, representative of the MTL) and ambient temperature ($\sim$20° C) (Fig. S1a). For this purpose, we used two techniques: the steady-state method and the pulse decay method ^7^. Each technique was employed on a different intact OPA sample cored parallel to the direction of bedding. Thus, two cylindrical samples of diameter and height of 38 mm were prepared.

Using Darcy’s law equation (Fig S1b), we have calculated permeability $k$ as:

| $\boldsymbol{k=}\frac{\boldsymbol{Q}}{\boldsymbol{A}}\left( \frac{\boldsymbol{\mu\cdot L}}{\boldsymbol{P}_{\boldsymbol{1}}\boldsymbol{-}\boldsymbol{P}_{\boldsymbol{2}}} \right)$ | (2) |
| --- | --- |

Where $P_{1}$ and $P_{2}$ corresponds to the pore pressures of reservoir 1 and 2, respectively, $\mathbf{Q}$ is the flux or total discharge (mm^3^/s), $A$ is the cross-sectional area and $L$ the length of the sample. The viscosity of water (T=25° C) $=8.9 x {10}^{-4} Pa s$. Following the pulse decay method (Figure 2c), we have estimated permeability $k$ as:

| $k=\frac{\alpha\cdot\mu\cdot\beta\cdot L}{A \cdot\left( \frac{1}{V_{1}}+\frac{1}{V_{2}} \right)}$ | (3) |
| --- | --- |

With

| $P_{1}-P_{f}=\Delta P\cdot\left( \frac{V_{2}}{V_{1}+V_{2}} \right)\cdot e^{-\alpha t}$ | (4) |
| --- | --- |

Where $V_{1}$ and $V_{2}$ are the volume of the reservoirs (including the volume of the connecting tubes), $\beta=4.6 x {10}^{-10} Pa^{-1}$ is water compressibility, $t$ is time, $\Delta P$ is the initial change in pore pressure and $P_{f}$ is the constant final pressure when the pore pressure equilibrium is reached. The change in pore pressure $\Delta P$ was set to be $\sim$5% of $P_{1}$ for every step-in confining pressure. The initial pulse was applied at the bottom of the sample, i.e., we changed $P_{1}$. The effective confining pressure ${P'}_{c}$ was therefore kept constant as $P_{c}-P_{1}\approx P_{c}-P_{2}$. Initial values of $P_{1}$ and $P_{2}$ were 2 MPa. The volumes $V_{1}$ and $V_{2}$ were initially $\sim$400.000 mm^3^ and $\sim$200.000 mm^3^, respectively.

The permeability of the two intact samples cored parallel to the bedding planes decreases from ~3 x 10^-19^ to ~4 x 10^-20^ m^2^ when the effective pressure increases from 2 to 12 MPa respectively (Fig. S2). If the data of the two samples are grouped, the permeability varied up to ~1 order of magnitude according to $\log\boldsymbol{k [}\boldsymbol{m}^{\boldsymbol{2}}\boldsymbol{]}\boldsymbol{= -0.05}\boldsymbol{P'}_{\boldsymbol{c}}\boldsymbol{-18.4}$, where $\boldsymbol{P'}_{\boldsymbol{c}}$ is the effective confining stress in MPa.


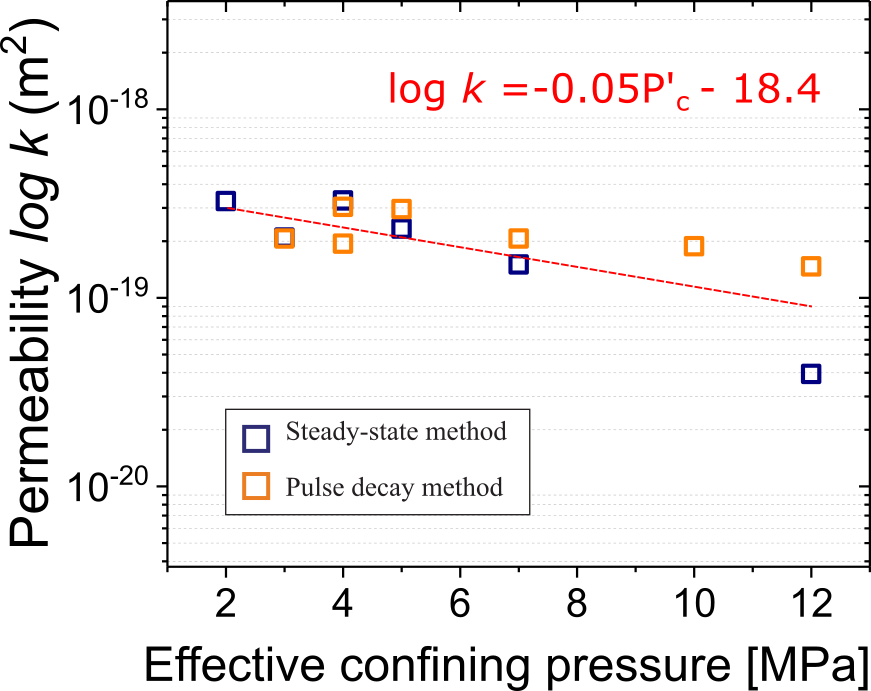


**Figure S2:** Permeability results from steady-state and pulse decay methods of two intact samples cored parallel to bedding. Permeability decreases when increasing effective confining stress. The logarithmic correlation for permeability parallel to the bedding $\log k= -0.05P_{c}^{'}-18.4$ is valid for all points.

The permeability values of intact OPA samples cored parallel to the bedding planes (10^-19^ to 10^-20^ m^2^) are in agreement with measured values in earlier laboratory studies^8–11^. We have not measured permeability perpendicular to bedding as we have focused on the capacity for fluid flow parallel to the direction of shearing. Permeability perpendicular to bedding is expected to be lower than permeability parallel to bedding, as shown by the same earlier studies cited in this section.

1. **Additional FIB-SEM images of the OPA fault gouge**

|  |  |
| --- | --- |
|  |  |
|  |  |

**Figure S3:** FIB-SEM image of the fault gouge of the Opalinus Clay formation.

1. **Porosity and grain density results**

**Table S1:** Porosity and grain density results

1. **Model of Yang & Aplin, (2010) based on an exponential law**

$$\ln(k[m^{2}])= a_{CF}+b_{CF}\cdot\frac{\emptyset}{1-\emptyset}+c_{CF}\cdot\left( \frac{\emptyset}{1-\emptyset} \right)^{0.5}$$

Where the coefficients $a_{CF}$, $b_{CF}$, and $c_{CF}$ are defined as:

$$a_{CF}=-69.59-26.79\cdot CF+44.07\cdot CF^{0.5}$$

$$b_{CF}=-53.61-80.03\cdot CF+132.78\cdot CF^{0.5}$$

$$c_{CF}=86.61+81.91\cdot CF-163.61\cdot CF^{0.5}$$

And $CF$ is the clay content (in fraction) and $\emptyset$ the related porosity.

**References**

1. Yang, Y. & Aplin, A. C. A permeability-porosity relationship for mudstones. *Mar. Pet. Geol.* **27**, 1692–1697 (2010).

2. Kübler, B. Cristallinité de l’illite, méthodes normalisées de préparations, méthodes normalisées de mesures. in *Cahiers de l’Institut de Géologie de Neuchâtel, Série ADX* vol. 1 13 (1987).

3. Adatte, T., Stinnesbeck, W. & Keller, G. Lithostratigraphic and mineralogic correlations of near K/T boundary clastic sediments in northeastern Mexico: Implications for origin and nature of deposition. in *The Cretaceous-Tertiary Event and Other Catastrophes in Earth History*  (eds. Ryder, G., Fastovsky, D. E. & Gartner, S.) (Geological Society of America, 1996).

4. Orellana, L. F., Scuderi, M. M., Collettini, C. & Violay, M. Do scaly clays control seismicity on faulted shale rocks? *Earth Planet. Sci. Lett.* **488**, 59–67 (2018).

5. Orellana, L. F., Scuderi, M. M., Collettini, C. & Violay, M. Frictional Properties of Opalinus Clay: Implications for Nuclear Waste Storage. *J. Geophys. Res. Solid Earth* **123**, 157–175 (2018).

6. Van Loon, L. R., Soler, J. M., Müller, W. & Bradbury, M. H. Anisotropic diffusion in layered argillaceous rocks: A case study with Opalinus Clay. *Environ. Sci. Technol.* **38**, 5721–5728 (2004).

7. Brace, W. F., Walsh, J. B. & Frangos, W. T. Permeability of granite under high pressure. *J. Geodyn.* **73**, 2225–2236 (1968).

8. Monfared, M., Sulem, J., Delage, P. & Mohajerani, M. Temperature and damage impact on the permeability of opalinus clay. *Rock Mech. Rock Eng.* **47**, 101–110 (2014).

9. T., P. *et al.* The effect of microstructural hetreogenity on pore size distribution and permeability in Opalinus Clay (MT- Switzerland): insights from an integrated study of laboratory fluid flow and pore morphology from BIB-SEM images. *Geol. Soc. London, Spec. Publ.* **454**, 85–106 (2017).

10. Senger, R., Romero, E. & Marschall, P. Modeling of Gas Migration Through Low-Permeability Clay Rock Using Information on Pressure and Deformation from Fast Air Injection Tests. *Transp. Porous Media* **123**, 563–579 (2018).

11. Yu, C. *et al.* Comparative study of methods to estimate hydraulic parameters in the hydraulically undisturbed Opalinus Clay (Switzerland). *Swiss J. Geosci.* **110**, 85–104 (2017).
